# Amniotic mesenchymal cells from pre‐eclamptic placentae maintain immunomodulatory features as healthy controls

**DOI:** 10.1111/jcmm.12715

**Published:** 2015-10-30

**Authors:** Stefano Pianta, Marta Magatti, Elsa Vertua, Patrizia Bonassi Signoroni, Ivan Muradore, Anna Maria Nuzzo, Alessandro Rolfo, Antonietta Silini, Federico Quaglia, Tullia Todros, Ornella Parolini

**Affiliations:** ^1^ Centro di Ricerca E. MenniFondazione Poliambulanza‐Istituto OspedalieroBresciaItaly; ^2^ Doctoral School of Translational and Molecular MedicineUniversity of MilanMilanItaly; ^3^ Department of Surgical SciencesO.I.R.M.‐S. Anna HospitalUniversity of TurinTurinItaly; ^4^ Department of Obstetrics and GynecologyFondazione Poliambulanza‐Istituto OspedalieroBresciaItaly

**Keywords:** amniotic mesenchymal stromal cells, placenta, pre‐eclampsia, immunomodulation, T reg, Th, CTL, phenotype, DC, macrophage

## Abstract

Pre‐eclampsia (PE) is one of the most severe syndromes in human pregnancy, and the underlying mechanisms of PE have yet to be determined. Pre‐eclampsia is characterized by the alteration of the immune system's activation status, an increase in inflammatory Th1/Th17/APC cells, and a decrease in Th2/Treg subsets/cytokines. Moreover, inflammatory infiltrates have been detected in the amniotic membranes of pre‐eclamptic placentae, and to this date limited data are available regarding the role of amniotic membrane cells in PE. Interestingly, we and others have previously shown that human amniotic mesenchymal stromal cells (hAMSC) possess anti‐inflammatory properties towards almost all immune cells described to be altered in PE. In this study we investigated whether the immunomodulatory properties of hAMSC were altered in PE. We performed a comprehensive study of cell phenotype and investigated the *in vitro* immunomodulatory properties of hAMSC isolated from pre‐eclamptic pregnancies (PE‐hAMSC), comparing them to hAMSC from normal pregnancies (N‐hAMSC). We demonstrate that PE‐hAMSC inhibit CD4/CD8 T‐cell proliferation, suppress Th1/Th2/Th17 polarization, induce Treg and block dendritic cells and M1 differentiation switching them to M2 cells. Notably, PE‐hAMSC generated a more prominent induction of Treg and higher suppression of interferon‐γ when compared to N‐hAMSC, and this was associated with higher transforming growth factor‐β1 secretion and PD‐L2/PD‐L1 expression in PE‐hAMSC. In conclusion, for the first time we demonstrate that there is no intrinsic impairment of the immunomodulatory features of PE‐hAMSC. Our results suggest that amniotic mesenchymal stromal cells do not contribute to the disease, but conversely, could participate in offsetting the inflammatory environment which characterizes PE.

## Introduction

Pre‐eclampsia is one of the main causes of maternal and foetal morbidity and mortality, causing nearly 40% of premature births delivered before 35 weeks of gestation, and complicating around 2–8% of all pregnancies worldwide 1, 2. The physiopathology triggering the disease is yet unknown, however, it is clear that the development of PE requires the presence of placenta, as the clinical syndrome does not develop in the absence of placenta and it disappears soon after birth. It is also widely accepted that the pathophysiological process of PE begins with an abnormal trophoblast invasion early in pregnancy, which produces increased placental oxidative stress contributing to the development of systemic endothelial dysfunction in the later phases of the disease 2.

Aberrant/chronic inflammation is considered to be a dominant component in the pathogenesis of PE. Indeed, in contrast to normal pregnancy characterized by Th2 type immunological state where Th2 and Treg cell responses and cytokine profiles predominate, in PE there is a predominance of Th1‐type immunity and pro‐inflammatory cytokines 3, 4, 5. In addition to the imbalance of Th1 and Th2 cells, increases of Th17 and reduction in Treg cells have also been found in maternal blood 6, 7, 8 and placenta 9. Although some authors did not find different numbers of Treg cells between pre‐eclamptic patients and women with uncomplicated pregnancies 10, 11, others have shown that the numbers of circulating Treg cells in PE are decreased compared to those in healthy pregnant women 12, 13. Moreover, the abnormal activation status of antigen presenting cells (APC) [specifically monocytes, macrophages and dendritic cells (DC)] was found in pre‐eclamptic placentae 14, 15. Indeed, in normal pregnancy, most of the macrophages are anti‐inflammatory/immunomodulatory M2 macrophages, while in PE, there appears to be increased numbers of inflammatory M1 macrophages 16.

The mechanisms underlying this aberrant inflammatory profile need to be elucidated. However, it is plausible that it arises from the lack of or altered immune regulation/modulation. Indeed, foetal–maternal tolerance, a fundamental process for a successful pregnancy, is thought to be altered in PE 17. Interestingly, increasing evidence supports the idea that mesenchymal stem/stromal cells (MSC), derived from both maternal and foetal compartments, strongly contribute to foetal–maternal tolerance, mainly because of their broad immune regulatory properties 18, 19, 20, 21, 22, 23. Within foetal MSC, the immune regulatory properties of MSC from human amniotic membrane (hAMSC) are obtaining growing interest 24, 25. We and others have demonstrated that hAMSC are able to modulate lymphocyte proliferation when cultured either in contact or non‐contact (transwell system) with lymphocytes, and even when the conditioned medium (CM) derived from the culture of unstimulated hAMSC was used instead of the cells 26, 27, 28, 29, 30. The use of CM and the transwell system demonstrated that hAMSC could act on activated lymphocytes through constitutively produced, inhibitory secreted factor(s), without the need for cell‐to‐cell contact or activating stimuli. We recently demonstrated that hAMSC reduce the expression of the markers associated with the Th1 and Th17 populations, and significantly induce the regulatory T‐cell compartment 30. Furthermore, others have shown that not only T lymphocytes but also the cytotoxicity of natural killer cells (NK) were affected by amniotic membrane‐derived cells 31. Moreover, we and others have shown that hAMSC also act on the monocytes suppressing their differentiation towards 32, 33, 34. Interestingly, hAMSC do not simply block the differentiation of monocytes, but skew differentiation into anti‐inflammatory M2 macrophage‐like cells 33.

Overall, these results suggest that hAMSC could regulate different immune cell types which are described to be dysregulated in PE. Moreover, lesions of amniotic basement membrane associated with inflammatory infiltrate, as well as inflammatory reaction of the amnion, have been found in PE 35, 36. Whether or not the immunomodulatory properties of hAMSC are altered in or contribute to the development of PE remains to be understood.

The aim of this study was to understand if hAMSC isolated from PE (PE‐hAMSC) pregnancies presented altered immunological features. To this end, we compared the immunomodulatory properties of PE‐hAMSC with those from normal/uncomplicated pregnancies (N‐hAMSC), both from the third trimester. We performed a comprehensive study of cell surface phenotype, ability to suppress T‐cell proliferation and to trigger their polarization towards CD4 Th1, Th2, Th17, Treg cells and CD8 cytotoxic T cells. Finally, we also compared their ability to affect monocyte differentiation towards DC, M1 and M2 macrophage‐like cells.

## Materials and methods

### Ethic statement

Healthy and pre‐eclamptic human placentae were collected after obtaining informed written consent according to the guidelines set by the Ethical Committee of the Catholic Hospital (CEIOC) (Document ‘*Parere 16/2012*') and Institutional Ethical Review Board of O.I.R.M. S.Anna Hospital, Turin and ‘Ordine Mauriziano di Torino' (n.209; protocol 39226/C.27.1 04/08/09), respectively.

### Isolation and culturing of hAMSC

Human placentae from both normal and pre‐eclamptic pregnancies were collected and processed immediately. Pre‐eclamptic placentae were selected according to previously described criteria 37. Human amniotic mesenchymal stromal cells were isolated as previously described 29. Both freshly isolated (p0) PE‐hAMSC and N‐hAMSC were sub‐cultured until passages 4 (p4) by plating at a density of 10^4^/cm^2^ in Chang medium^®^ C (Irvine Scientific, Santa Ana, CA, USA) supplemented with 2 mM L glutamine. The study was performed with PE‐hAMSC at gestational age of 31.5 ± 2.6 (*n* = 6) and, given the difficulty in obtaining healthy/normal placentae from preterm pregnancies, we used N‐hAMSC from term placentae (*n* = 6, gestational age = 38.8 ± 0.44) (Table 1).

**Table 1:** Clinical features of the study population

	Healthy (*n* = 6)	Pre‐eclampsia (*n* = 6)	*P*‐value
Nulliparae (%)	33	50	n.s.
Gestational age at delivery (weeks, median – range)	38.5 (38–39)	31.6 (28–35)	<0.001
Maternal age at delivery (years, median – range)	31 (25–38)	35.5 (33–46)	n.s.
Caucasian ethnicity (%)	100	100	n.s.
Smokers (%)	0	0	n.s.
Alcohol (%)	0	0	n.s.
Previous prenatal admission (%)	16.7	50	
Systolic Blood pressure (mmHg, median – range)	112.5 (90–140)	164 (155–170)	<0.001
Diastolic blood pressure (mmHg, median – range)	70 (60–80)	99.5 (90–104)	<0.001
Proteinuria (g/24 hr, median – range)	Absent	6.4 (0.86–15)	<0.05
A/REDF (%)	0	17	n.s.
Pathological uterine Doppler (%)	0	83	<0.05
Labour (%)	50	17	n.s.
Caesarean section (%)	66.7	83	n.s.
Birth weight (g, median – range)	2922.5 (2375–3750)	1307.5 (880–2500)	<0.001
Placental weight (g median – range)	590 (450–600)	225.5 (150–395)	<0.001
Foetal sex				
Male (%)	33	33	n.s.
Female (%)	67	67	n.s.

n.s.: not significant.

### hAMSC phenotype analysis

Surface phenotype of N‐ and PE‐hAMSC at p4 were investigated by flow cytometry following standard protocols as previously reported 33. Cells were acquired with a FACSCalibur (BD Biosciences, San Jose, CA, USA) and analysed with FCS express v4.07 (DeNovo Software, Los Angeles, CA, USA). Dead cells were gated out by propidium iodide staining (0.1 μg/ml; Sigma‐Aldrich, St Louis, MO, USA). Antibodies and suppliers used are described in Table 2.

**Table 2:** Antibodies used for flow cytometry analysis

hAMSC phenotype	
Mesenchymal stromal cell markers	CD10 (Neprilysin) FITC, CD13 (Aminopeptidase N) PE, CD26 (Dipeptidyl peptidase 4) FITC, CD44 (CD44 antigen) FITC, CD73 (5′‐NT) PE, CD90 (Thy‐1) FITC, CD105 (Endoglin) FITC, CD106 (VCAM‐1) PE, CD109 (CD109 antigen) PE, CD140b (PDGFR‐beta) PE, CD146 (MUC18) PE, CD166 (ALCAM) PE, CD200 (OX20) PE, CD271 (NFGR) APC
Co‐stimulatory molecules	B7‐H4 APC, CD59 (CD59 glycoprotein) FITC, CD70 (CD70 antigen) FITC, CD85a (LIR‐3) Alexa Fluor^®^ 488, CD152 (CTLA‐4) APC, CD154 (CD40L) FITC, CD244 (2B4) FITC, CD252 (OX40‐L) PE, CD272 (BTLA) PE, CD273 (PD‐L2) APC, CD274 (PD‐L1) PE, CD275 (ICOS ligand) PE, CD282 (Toll‐like receptor 2) FITC, CD284 (Toll‐like receptor 4) PE, CD357 (AITR/GITR) APC^a^
Haematopoietic markers	CD33 (Siglec‐3) FITC, CD34 (Haematopoietic progenitor cell antigen CD34) FITC, CD45 (L‐CA) APC
Human leucocyte antigens class I and II	HLA‐ABC FITC, HLA‐DM PE, HLA‐DQ FITC, HLA‐DR APC, HLA‐G FITC^b^
Cytokine receptors	CD114 (G‐CSFR) PE, CD116 (GM‐CSFR) PE, CD119 (IFN‐gamma receptor 1) PE, CD120b (TNF‐RII) PE, CD124 (IL‐4Ra) PE
Integrins and adhesion molecules	CD49a (Integrin alpha‐1) Alexa Fluor^®^ 488, CD49b (Integrin alpha‐2) FITC, CD49c (Integrin alpha‐3) PE, CD49d (Integrin alpha‐4) PE
Others	CD31 (PECAM‐1) FITC, CD39 (NTPDase 1) PE‐Cy7^a^, CD52 (CAMPATH‐1) FITC, CD117 (c‐kit) APC, SSEA‐4 PE
T‐cell subset analysis	
Th1	CD4 BV421/BV510/BB515, CD45RA FITC/PerCP‐Cy^™^5.5, CD183 (CXCR3) PE‐Cy^™^7, IFN‐γ PE‐CF594, TNFα PE‐Cy7
Th2	CD4 BV421/BV510/BB515, CD45RA FITC/PerCP‐Cy^™^5.5, CD294 (Prostaglandin D2 receptor 2) PE‐CF594, IL‐4 BV421, IL‐13 PE
Th17	CD4 BV421/BV510/BB515, CD45RA FITC/PerCP‐Cy^™^5.5, CD161 (NKR‐P1A) Alexa Fluor^®^ 647, IL‐17A PE, IL‐17F Alexa Fluor^®^ 647
Treg	CD4 BV421/BV510/BB515, CD45RA FITC/PerCP‐Cy^™^5.5, CD25 (IL‐2‐RA) PerCP‐Cy^™^5.5, FoxP3 PE‐CF594, TGF‐β1 BV421
CTL	CD8 BV510/Alexa Fluor^®^ 647, CD45RA FITC/PerCP‐Cy^™^5.5, Granzyme B PE‐CF594, TNFα PE‐Cy7
Monocytes analysis	
DC/macrophage markers	CD1a (T‐cell surface glycoprotein CD1a) FITC, CD14 (Monocyte differentiation antigen CD14) PE‐Cy7, CD83 (CD83 antigen) PE, CD163 (Scavenger receptor cysteine‐rich type 1 protein M130) BV421, CD197 (C‐C chemokine receptor type 7) Alexa Fluor^®^ 647, CD209 (DC‐SIGN) PerCP‐Cy5.5
Co‐stimulatory molecules	CD40 (Tumour necrosis factor receptor superfamily member 5) FITC, CD80 (T‐lymphocyte activation antigen CD80) FITC, CD273 (PD‐L2) APC, CD274 (PD‐L1) BV421

a eBiosciences, San Diego, CA, USA.

b Bio‐Rad AbD Serotec, Raleigh, NC, USA.

All antibodies were purchased from BD Biosciences, unless otherwise specified.

### Isolation of peripheral blood mononuclear cells, T cells, and monocytes

Peripheral blood was collected from healthy adult donors. Human peripheral blood mononuclear cells (PBMCs) were obtained by density gradient centrifugation (Histopaque; Sigma‐Aldrich) of buffy coats. PBMC were γ‐irradiated (30 Gy) prior to use as allogeneic stimulators. T lymphocytes and monocytes were purified from PBMC by using Pan T cell Isolation Kit II and anti‐CD14‐coated microbeads, respectively, according to the manufacturer's instructions (both from Miltenyi Biotec, Bergisch Gladbach, Germany).

### Mixed lymphocyte culture

Co‐cultures of T cells with N‐ and PE‐hAMSC were established in direct cell‐to‐cell contact. Around 10^5^ hAMSC were seeded in 96‐well plates (Nunc, Roskilde, Denmark) in 150 μl of UltraCulture medium (Lonza, Basel, CH, Switzerland) and irradiated (30 Gy) to block proliferation. The day after, mixed lymphocyte cultures (MLC) were obtained by culturing 10^5^ T lymphocytes and 10^5^ γ‐irradiated allogeneic PBMC in 100 μl of UltraCulture medium (Lonza), in the absence (controls) or presence of hAMSC. T‐cell proliferation was assessed by 5‐ethynyl‐2′deoxyuridine (EdU) incorporation as previously described 38. Briefly, 10 μM EdU (Life Technologies, Carlsbad, CA, USA) was added on day 5 and incubated for 16–18 hrs. Incorporated EdU was detected by the Cu‐catalysed alkyne‐azide cycloaddition (CuAAC or ‘click') reaction of the ethynyl group with 2.5 μM 3‐azido‐7‐hydroxycoumarin (Jena Biosciences, Jena, Germany), in buffer solution (100 mM Tris‐HCl pH 8.0, 10 mM L‐ascorbic acid, 2 mM CuSO_4_) at RT for 30 min. The samples were acquired with a FACSAria (BD Biosciences) and analysed with FCS express v4.07 (DeNovo Software).

### Analysis of different T‐cell subsets

The phenotypes of different T‐cell subsets derived from MLC experiments were assessed after 6 days of co‐culturing by FACS analysis using a set of cell surface markers and intracellular cytokines to characterize CD4^+^ T helper (Th) cells Th1 39, 40, Th2 39, 40, Th17 39, 40, Treg 41 and CD8^+^ cytotoxic T lymphocytes (CTL) 42, as reported in Table 2. Before fixation, samples were stained with Zombie NIR Live/Dead Cell Kit to remove dead cells from the analysis (eBiosciences, San Diego, CA, USA). Cells were then fixed, stained for surface antigens, permeabilized and stained for intracellular antigens as reported in 30. In parallel, cells from MLC experiments were also evaluated for T‐cell intracellular cytokine production. Therefore, cells were stimulated for 5 hrs at 37°C with phorbol‐12‐myristate‐13‐acetate (50 ng/ml), calcium‐ionomycin (CaI, 1 μg/ml) and Brefeldin A (10 μg/ml) (all from Sigma‐Aldrich), and viable cells were analysed on a flow cytometer.

### Detection of secreted cytokines

The supernatant from MLC experiments was collected 2 and 6 days after culturing. Quantification of secreted interferon (IFN)‐γ, interleukin (IL)‐1‐β, IL‐2, IL‐5, IL‐9, IL‐10, IL‐13, IL‐22, sIL‐2R, tumour necrosis factor (TNF)‐α and transforming growth factor (TGF)‐β was evalua‐ted by using a multiple cytometric bead array system FlowCytoMix (eBiosciences), according to the manufacturer's instructions. Samples were acquired with a FACSAria (BD Biosciences) and analysed with FlowCytomix Pro software (eBiosciences).

### Generation of monocyte‐derived DC, M1 and M2 macrophage‐like cells

Dendritic cells were obtained by culturing 1 × 10^6^ CD14^+^ monocytes as previously described 33. To obtain the herein termed M1 and M2 macrophage‐like cells, 0.5 × 10^6^ CD14^+^ monocytes were cultured for 4 days with 5 ng/ml human recombinant Granulocyte macrophage‐colony stimulating (GM‐CSF) (Miltenyi Biotec) or 20 ng/ml M‐CSF (Miltenyi Biotec) respectively. M1 were then treated with 20 ng/ml IFN‐γ (BD Biosciences) and 100 ng/ml Lipopolysaccharide (LPS) (Sigma‐Aldrich), whereas M2 with 20 ng/ml IL‐4 (by Dr. Schweighoffer, Novartis, Vienna, Austria) and 100 ng/ml LPS (Sigma‐Aldrich), for 2 days. All cultures were performed in 24‐well plates (Corning, Corning, NY, USA) at 37°C in 1 ml of Roswell Park Memorial Institute (RPMI) complete medium.

### Exposure of monocytes to hAMSC

Monocytes were differentiated towards DC, M1‐ and M2 macrophage‐like cells in the absence (controls) or presence of normal and PE hAMSC, either in direct cell‐to‐cell contact or with physical separation using transwell chambers. hAMSC were seeded in 24‐well plates in 0.5 ml (for contact experiments) or in 0.3 ml of RPMI complete medium in the upper compartment of a transwell (0.4 μm pore, polycarbonate membrane; Corning) and then irradiated (30 Gy). The day after, monocytes in RPMI complete medium were added into the same well (contact experiments) or into the lower compartment of 24‐well plates (transwell experiments) at a monocyte: hAMSC ratio of 1:0.05 (contact experiments) or 1:0.4 (transwell experiments). Dendritic cells and macrophage differentiation was carried out with appropriate stimuli, as described above.

### Monocytes phenotype analysis

Monocytes were differentiated towards DC, M1‐ and M2 macrophage‐like cells in the absence or presence of hAMSC (as described above). After culturing, the monocytes were collected by gently scraping cells from the wells. Surface phenotype was investigated by flow cytometry as previously reported 33, using cell markers described to be expressed by DC 43, 44, M1‐ 45 and M2 45, 46 macrophages. Cells were acquired with a FACSAria (BD Biosciences) and analysed with the FCS express v4.07 (DeNovo Software). Antibodies and suppliers used in this study are described in Table 2.

### Statistical analysis

**Figure 1:**
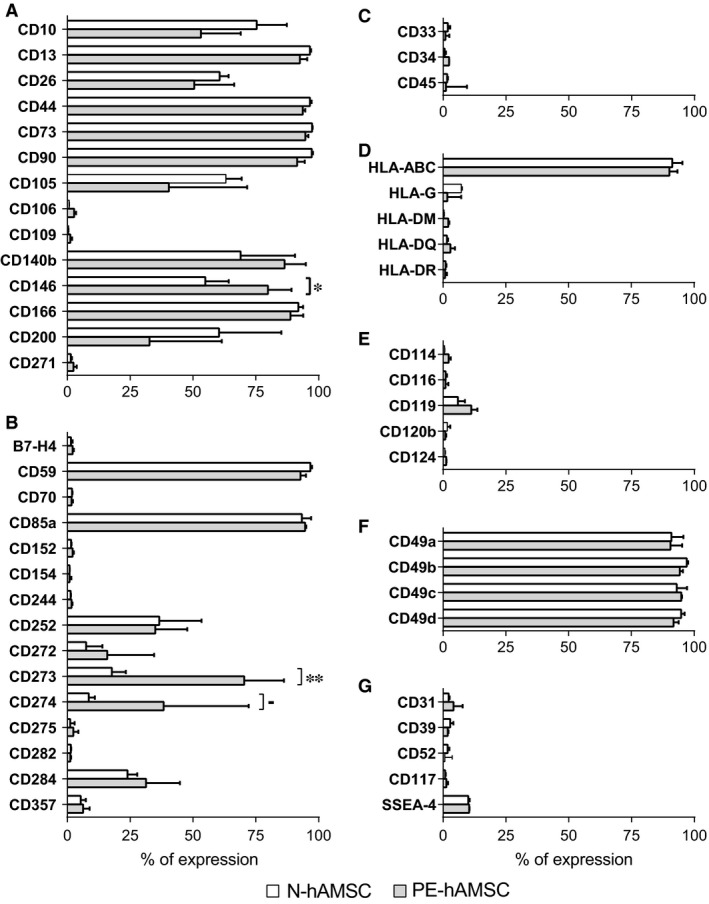
Immunophenotype of N‐hAMSC and PE‐hAMSC. N‐hAMSC (*n* = 4) (□) and PE‐hAMSC (*n* = 5) (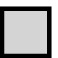) at p4 were analysed by flow cytometry for markers described to be expressed by MSC (**A**), co‐stimulatory molecules (**B**), haematopoietic markers (**C**), human leucocyte antigens (HLA) class I and class II (**D**), cytokine receptors (**E**), integrins and adhesion molecules (**F**), and others (**G**). ^‐^ *P* = 0.07; *P < 0.05; **P < 0.01.

Unless otherwise indicated, the results were obtained from six independent experiments, and are represented as median and interquartile range. Statistical analysis was performed by both parametric and non‐parametric approaches. Comparison between two groups (results shown in Table 1 and Fig. 1) was performed by unpaired *t*‐test and Mann–Whitney test. For all the other results, statistical analysis was performed by one‐way anova followed by the Bonferroni post hoc test, and Kruskal–Wallis anova by ranks. Analyses were performed with GraphPad Prism 6 software (GraphPad Software, San Diego, CA, USA). A *P*‐value lower than 0.05 was considered statistically significant.

## Results

### Phenotype analysis of N‐hAMSC and PE‐hAMSC

N‐hAMSC and PE‐hAMSC were cultured up to passage 4 (P4). Both N‐hAMSC and PE‐hAMSC formed a monolayer of fibroblast‐like cells with the typical mesenchymal stromal morphology without visible morphological differences (data not shown).

We analysed the phenotype of cells at P4 for the expression of selected markers (Fig. 1). These include those described to be expressed by MSC (Fig. 1A), co‐stimulatory molecules (Fig. 1B), haematopoietic markers (Fig. 1C), human leucocyte antigens (HLA) class I and class II (Fig. 1D), cytokine receptors (Fig. 1E), integrins and adhesion molecules (Fig. 1F), and others (Fig. 1G).

Flow cytometry analysis revealed that the surface marker profile of PE‐hAMSC was similar to that of N‐hAMSC. Specifically, both PE‐hAMSC and N‐hAMSC expressed all the MSC markers examined, with the exception of CD106, CD109 and CD271 (Fig. 1A). Furthermore, they expressed different integrins and adhesion molecules (Fig. 1F). Moreover, they expressed HLA‐ABC (HLA class I), but lacked HLA‐G (non‐classical HLA class I) and HLA‐DM, ‐DQ, ‐DR (HLA class II) (Fig. 1D). They were negative (or weakly positive) for haematopoietic markers (Fig. 1C), cytokine receptors (Fig. 1E), and CD31, the ectonucleotidase CD39, and CD52 (Fig. 1G). Among the co‐stimulatory molecules tested, they expressed CD59, CD85a, CD252, CD272, CD273, CD274 and CD284, and were negative for the other molecules investigated (Fig. 1B).

Even though PE‐hAMSC and N‐hAMSC had similar profiles, several quantitative differences were found. The expression of CD146 and of co‐stimulatory molecule CD273 (PD‐L2) was significantly higher in PE‐hAMSC than in N‐hAMSC (Fig. 1A and B). Similarly, the expression of CD274 (PD‐L1) was higher in PE‐hAMSC when compared to N‐hAMSC, although the difference did not reach statistical significance (Fig. 1B).

### The effects of N‐hAMSC and PE‐hAMSC on T‐cell proliferation and polarization

**Figure 2:**
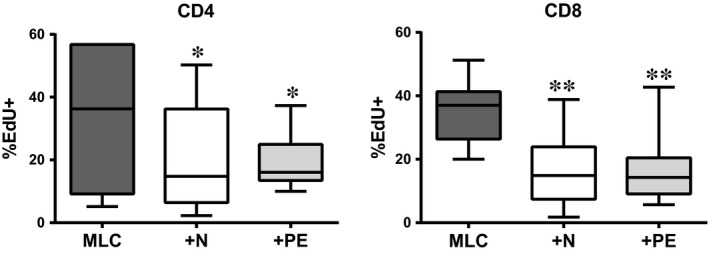
Effects of N‐hAMSC and PE‐hAMSC on the proliferation of CD4 and CD8. T cells were stimulated with allogeneic PBMC in mixed lymphocyte culture in the absence (MLC, 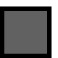) or presence of N‐hAMSC (+N, □) and PE‐hAMSC (+PE, 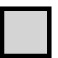). The box‐plots show CD4 and CD8 proliferation expressed as percentage (%) of EdU‐positive cells. *P < 0.05; **P < 0.01.

We have previously demonstrated that hAMSC, either freshly isolated (passage 0) or after cell expansion, are able to modulate lymphocyte proliferation 27. Herein, we compared the effects of hAMSC derived from normal and pre‐eclamptic placentae on the proliferation of CD4^+^ and CD8^+^ lymphocytes. As shown in Figure 2, both N‐ and PE‐hAMSC suppressed the proliferation of both CD4^+^ Th cells and CD8^+^ CTL.

**Figure 3:**
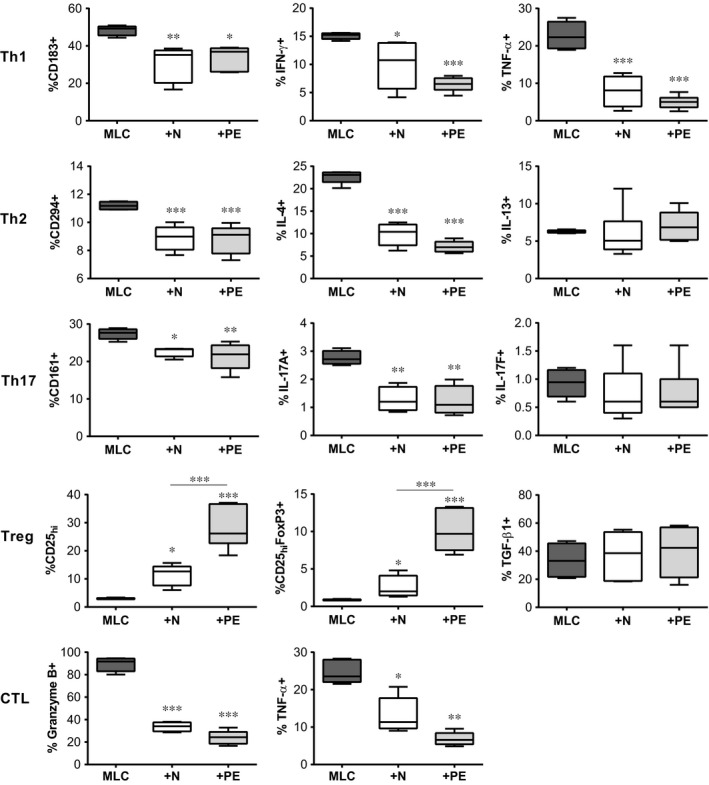
PE‐hAMSC modulate CD4 and CD8 lymphocytes inducing a strong shift towards the Treg pathway. Flow cytometry analysis of the different T‐cell subsets was performed after 6 days of co‐culturing on allostimulated T cells in the absence (MLC, 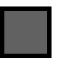) or presence of N‐hAMSC (+N, □) and PE‐hAMSC (+PE, 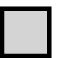). The plots represent the percentage of CD4^+^ CD183^+^, CD4^+^ IFN‐γ, CD4^+^ TNF‐α (Th1 panel), CD4^+^ CD294^+^, CD4^+^ IL‐4^+^, CD4^+^ IL‐13^+^ (Th2 panel), CD4^+^ CD161^+^, CD4^+^ IL‐17A^+^, CD4^+^ IL‐17F^+^ (Th17 panel), CD4^+^ CD25_high_, CD4^+^ CD25_high_FoxP3^+^, CD4^+^ TGF‐β1 (Treg panel), CD8^+^Granzyme B^+^, CD8^+^ TNF‐α^+^ (CTL panel). All the populations were gated on the live CD45RA ^−^ fraction. *P < 0.05; **P < 0.01; ***P < 0.001.

To assess the effects of N‐hAMSCs and PE‐hAMSCs on the polarization of T cells, we evaluated the phenotype of CD4^+^ (Th and Treg) and CD8^+^ (cytotoxic) cells using different surface and intracellular markers. As shown in Figure 3 (Th1 panel), both N‐hAMSC and PE‐hAMSC significantly reduced the percentage of markers associated to Th1 cells, such as CD183^+^ (CXCR3), IFN‐γ+ and TNF‐α+. Furthermore, co‐culturing with both N‐hAMSC and PE‐hAMSC significantly reduced the percentage of cells positive for markers associated with the Th2 population, such as CD294 (CRTH2) and IL‐4, however, no changes were observed in the percentage of cells expressing IL‐13 (Fig. 3, Th2 panel). Regarding Th17 population, both N‐hAMSC and PE‐hAMSC reduced the percentage of cells positive for CD161 (KLRB1) and IL‐17A, whereas no significant changes were observed in the percentage of CD4^+^ expressing IL‐17F (Fig. 3, Th17 panel). Finally, we observed that both N‐hAMSC and PE‐hAMSC significantly modulated the T regulatory (Treg) compartment (Fig. 3, Treg panel) identified as CD4^+^ CD45RA‐CD25high and CD4^+^ CD45RA‐CD25highFoxP3^+^ cells. Both N‐hAMSC and PE‐hAMSC significantly increased CD25high and CD25highFoxP3^+^ cells, and interestingly, PE‐hAMSC to a greater extent than N‐hAMSC. Neither N‐hAMSC nor PE‐hAMSC were able to significantly alter the percentage of TGF‐β‐expressing CD4^+^ cells.

To investigate the effects of N‐hAMSC and PE‐hAMSC on CD8 CTL, we evaluated intracellular GranzymeB and TNF‐α expressed on CD8^+^ cells after co‐culturing with either normal or pre‐eclamptic cells. We observed that both N‐hAMSC and PE‐hAMSC intensively modulate CTLs by reducing GranzymeB and TNF‐α‐expressing cells (Fig. 3, CTL panel). Taken together, we show that both N‐hAMSC and PE‐hAMSC are able to inhibit the proliferation of alloreactive T lymphocytes and down‐regulate the Th pathway in a similar manner. Both normal and pre‐eclamptic cells can significantly enhance the Treg compartment, but pre‐eclamptic cells to a greater extent.

### The effects of N‐hAMSC and PE‐hAMSC on secreted cytokines

To provide further insight into which T‐cell subsets were altered by N‐hAMSC and PE‐hAMSC, we studied T‐cell cytokine profiles 2 and 6 days after co‐culturing with normal or pre‐eclamptic cells. The panel of cytokines analysed for the different T‐cell subsets are as follows: IFN‐γ, TNF‐α, IL‐1β, IL‐2, IL‐5, IL‐9, IL‐10, IL‐13, IL‐22, TGF‐β1, sIL‐2R.

**Figure 4:**
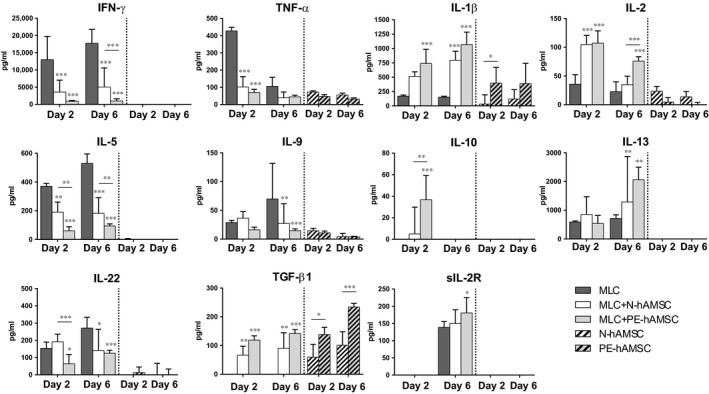
Effects of N‐hAMSC and PE‐hAMSC on T‐cell cytokine secretion. The secretion of IFN‐γ, IL‐1‐β, IL‐2, IL‐5, IL‐9, IL‐10, IL‐13, IL‐22, sIL‐2R, TNF‐α and TGF‐β, was evaluated 2 and 6 days after co‐culturing in the absence (MLC, 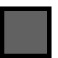) or presence of N‐hAMSC (MLC+N‐hAMSC, □) and PE‐hAMSC (MLC+PE‐hAMSC, 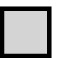). The amount of cytokine produced by N‐hAMSC (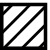) and PE‐hAMSC (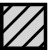) alone is represented on the right side of each plot. *P < 0.05; **P < 0.01.

N‐hAMSC and PE‐hAMSC significantly inhibited the secretion of pro‐inflammatory Th1‐cytokines TNF‐α and IFN‐γ, 2 and 6 days after co‐culturing (Fig. 4). Six days after co‐culturing, the inhibition of IFN‐γ was stronger with PE‐hAMSC (*P* < 0.05) compared to that seen with N‐hAMSC. Interestingly, IL‐1β secretion increased over time in the presence of N‐hAMSC and PE‐hAMSC, even if PE‐hAMSC released more IL‐1β *per se* (Fig. 4). The presence of N‐hAMSC or PE‐hAMSC induced the secretion of IL‐2 at day 2 of co‐culturing, and this induction decreased at day 6, but remained higher in the presence of PE‐hAMSC. The secretion of IL‐5, a Th2‐related cytokine, was markedly reduced by the presence of N‐hAMSC and PE‐hAMSC, but PE‐hAMSC induced a stronger inhibition of IL‐5 at day 6 (*P* < 0.05). Conversely, 6 days after co‐culturing with either N‐hAMSC or PE‐hAMSC, we observed an increase in the secretion of IL‐13 (Fig. 4). The release of Th17‐ related cytokine IL‐22 and Th9‐related cytokine IL‐9 were inhibited by both N‐hAMSC and PE‐hAMSC, and the reduction observed in the presence of PE‐hAMSC reached significance after 6 days of co‐culturing (*P* < 0.05, Fig. 4). The presence of N‐hAMSC or PE‐hAMSC induced the secretion of IL‐10 starting 2 days after co‐culturing, and this induction was greater in the presence of PE‐hAMSC (*P* < 0.05), but completely lost after 6 days (Fig. 4). TGF‐β1 was significantly higher in the presence of N‐hAMSC or PE‐hAMSC, but this effect is likely because of the amount of TGF‐β1 secreted by hAMSC (both N‐ and PE‐) *per se*. Of note, both 2 and 6 days after co‐culturing PE‐hAMSC released more TGF‐β1 compared to N‐hAMSC. Finally, sIL‐2R secretion significantly increased in the presence of N‐hAMSC or PE‐hAMSC after 6 days, and PE‐hAMSC induced a stronger release of sIL‐2R (Fig. 4).

### Impact of N‐hAMSC and PE‐hAMSC on the differentiation of monocytes towards DC, M1 and M2 macrophage‐like cells

**Figure 5:**
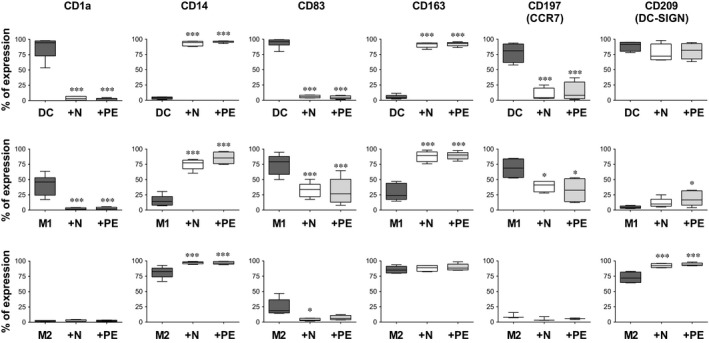
Effects of N‐hAMSC and PE‐hAMSC on the differentiation of monocytes towards DC, M1 and M2 macrophage‐like cells. Flow cytometry analysis of monocytes differentiated towards DC (upper panels), M1‐ (middle panels) and M2 macrophage‐like cells (lower panels) in the absence (controls, 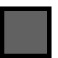) or presence of N‐hAMSC (□) and PE‐hAMSC (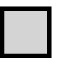). The box‐plots show the percentage (%) of positive cells for each marker. *P < 0.05, ***P < 0.001.

**Figure 6:**
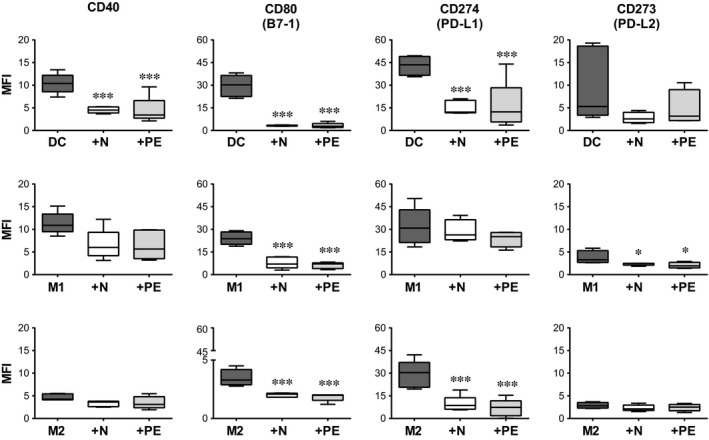
Co‐stimulatory molecule expression by monocytes differentiated towards DC, M1 and M2 macrophage‐like cells in the presence of N‐hAMSC and PE‐hAMSC. Flow cytometry analysis of monocytes differentiated towards DC (upper panels), M1‐ (middle panels), and M2 macrophage‐like cells (lower panels) in the absence (controls, 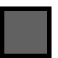) or presence of N‐hAMSC (□) and PE‐hAMSC (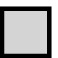). The box‐plots show the median fluorescence intensity (MFI) of each marker. *P < 0.05, ***P < 0.001.

Because of the important role of the activation status of antigen presenting cells in the pathophysiology of PE 16, we compared the effects of N‐hAMSC or PE‐hAMSC on the differentiation of peripheral blood CD14^+^ monocytes towards DC, M1‐, and M2 macrophage‐like cells. This was performed in the absence or presence of N‐hAMSC or PE‐hAMSC, either in direct contact with monocytes or with physical separation using a transwell system. After differentiation, cells were analysed using a panel of surface markers and co‐stimulatory molecules to discriminate between DC, M1, and M2‐derived macrophages. As shown in Figures 5 and 6, DC were characterized by the expression of CD1a, CD209 (DC‐SIGN), activation marker CD197 (chemokine receptor CCR7) and CD83, and co‐stimulatory molecules CD40, CD80, CD274 (PD‐L1) and CD273 (PD‐L2), whereas they lacked CD14 and CD163. Similar to DC, M1 macrophage‐like cells were characterized by a higher expression of activation and co‐stimulatory molecules such as CD197, CD83, CD40, CD80, CD274 (PD‐L1) and CD273 (PD‐L2) (Figs 5 and 6) and low/absent expression of CD14, and CD163 (Figs 5 and 6). However, compared to DC, M1 macrophage‐like cells were negative for CD209 and showed an intermediate expression of CD1a (Fig. 5). On the other hand, M2 macrophage‐like cells expressed CD14, CD163 and CD209 (Fig. 5). When compared to DC and M1, M2 macrophage‐like cells expressed reduced levels of CD40, CD80 (Fig. 6) and were negative for CD1a, CD83 and CD197 (Fig. 5).

When monocytes were differentiated towards DC in the presence of hAMSC, we observed that both N‐hAMSC and PE‐hAMSC induced the complete inhibition of DC differentiation. Indeed, these monocytes failed to express CD1a, but highly expressed CD14 and CD163 (Fig. 5). Moreover, the activation of the co‐stimulatory molecules CD197, CD83, CD40, CD80 and CD274 (PD‐L1) were highly suppressed (Figs 5 and 6). These effects were observed in both contact (Figs 5 and 6) and transwell settings (data not shown), without significant differences. Similarly, when monocytes were differentiated under M1 culture conditions in the presence of either N‐hAMSC or PE‐hAMSC, we observed the same inhibitory effect without significant differences. Specifically, they highly expressed CD14, CD163 and had increased CD209 expression (Fig. 5). Furthermore, when differentiated in the presence of N‐hAMSC and PE‐hAMSC, we observed a reduced expression of CD1a, CD197, CD83, CD80 and CD273 (PD‐L2) (Figs 5 and 6), whereas the expression of CD40 and CD274 (PD‐L1) did not change compared to control M1 macrophage‐like cells (Fig. 6). Overall, the expression of CD14, CD163 and CD209 combined with the absence/reduced expression of CD1a, CD197, CD83 and CD80 indicate that the differentiation of monocytes towards DC and M1 macrophage‐like cells was not only inhibited, but switched towards M2‐like cells.

Instead, when monocytes were treated under M2 conditions in the presence of either N‐hAMSC or PE‐hAMSC, their phenotype was almost unchanged. They expressed the same markers of control M2 macrophage‐like cells, but CD14 and CD209 to a higher extent (Fig. 5), and CD83, CD80 and CD274 to a lower extent (Figs 5 and 6). In the non‐contact setting (transwell system), the expression of CD40 was also reduced (data not shown).

## Discussion

In this study, we investigated the *in vitro* immunomodulatory properties of hAMSC isolated from pre‐eclamptic pregnancies (PE‐hAMSC), and compared them to hAMSC from the placentae of normal, uncomplicated pregnancies (N‐hAMSC). To our knowledge, this is the first study demonstrating that PE‐hAMSC maintain the same immunomodulatory features as N‐hAMSC. Notably, PE‐hAMSC generated a more prominent induction of Tregs, when compared to N‐hAMSC, and similar to N‐hAMSC, were able to block monocyte differentiation towards DC and M1.

Pre‐eclampsia is one of the most severe syndromes in human pregnancy, leading to maternal and neonatal morbidity and mortality, but the physiopathology that triggers the disease is still not understood. During PE, alteration of the activation status of the immune system occurs, and specifically, PE is characterized by the increase in inflammatory Th1/Th17/APC and decrease in Th2/Treg cytokines/subsets. These alterations have been reported in systemic (maternal blood) levels 6, 7, 8, and locally in the maternal–foetal interface (decidua and trophoblast) 9, 15. However, inflammatory infiltrates were also found in other foetal compartments of pre‐eclamptic placentae, such as the chorionic villi and amniotic membrane 35, 36.

Nevertheless, in PE, limited data are available regarding foetal tissues, specifically on amniotic membranes. Interestingly, within the amniotic membrane, hAMSC have been described to possess broad anti‐proliferative and anti‐inflammatory properties towards almost all immune cells whose phenotype, activation status or distribution were described to be altered in PE. Whether alterations of hAMSC's immunomodulatory properties may be present in PE remain unknown, and comparing the immunomodulatory properties of hAMSC from normal and pre‐eclamptic pregnancies might provide insight into this disorder.

For the most part, and in line with other reports 47, 48, our studies show that the phenotype of PE‐hAMSC is similar to that of N‐hAMSC. Interestingly, we observed a significant increase in CD146 (MUC18) expression which could be related to the higher TGF‐β1 production in PE‐hAMSC when compared to N‐hAMSC. Accordingly, previous reports have shown that TGF‐β1 increases the expression of CD146 on MSC 49. Furthermore, we also observed a higher expression of CD273 (PD‐L2) and CD274 (PD‐L1) in PE‐hAMSC. This could be related to the inflammatory environment from which PE‐hAMSC derive, in line with previous reports showing that IFN‐γ inflammatory priming increases PD‐L2 and PD‐L1 expression in hAMSC 50. However, we cannot exclude that these phenotype differences could be because of the different gestational age of PE‐hAMSC (31.6 ± 2.6 weeks) compared to N‐hAMSC (38.8 ± 0.5 weeks). In fact, others have shown that gestational age can impact the phenotype of epithelial cells from the amniotic membrane of ovine placentae 51.

Next, we investigated the immunomodulatory features hAMSC and demonstrate that PE‐ hAMSC retain similar, and even in some cases enhanced, immunomodulatory properties than N‐hAMSC. Indeed, both PE‐hAMSC and N‐hAMSC suppress both CD4 and CD8 T‐cell proliferation, at passage 0 (data not shown) as well as at passage 4. They also were able to reduce the expression of GranzymeB and TNF‐α in CD8 cytotoxic T cells. Within the CD4 population, PE‐hAMSC reduced the expression of markers associated to Th1, Th2 and Th17 populations, as well as their specific subset‐related cytokines, such as TNF‐α, IFN‐γ, IL‐1β (Th1‐related), IL‐5 (Th2), IL‐9 (Th9) and IL‐22 (Th17), and, remarkably, the suppression of IFN‐γ and IL‐5 was higher in the presence of PE‐hAMSC than N‐hAMSC. Of note, PE‐hAMSC were more capable of increasing the percentage of Tregs. In line with these results, we found more IL‐2 and IL‐10 secretion in T cells co‐cultured with PE‐hAMSC than those co‐cultured with N‐hAMSC. Both cytokines have been described to be important for Treg survival and suppressive functions 52, 53. Interestingly, we found more secretion of TGF‐β1 and higher expression of PD‐L1 and PD‐L2 in PE‐hAMSC than N‐hAMSC. The increased level of TGF‐β1 has been also previously reported in maternal serum 54, 55 and placental tissues 37, 56, 57 from pre‐eclamptic patients. Moreover, TGF‐β1, PD‐L1 and PD‐L2 have been described to inhibit T‐cell receptor signalling, T‐cell proliferation, production of cytokines such as IFN‐γ and TNF‐α, and to regulate the generation of peripheral Tregs and the suppression of effector T cells 58, 59. Thus, the higher secretion of TGF‐β1 and higher expression of PD‐L2 and PD‐L1 found in PE‐hAMSC could explain the higher suppression of IFN‐γ and higher induction of Treg observed with PE‐hAMSC. Finally, we also show that PE‐hAMSC not only act on T cells but are also able to inhibit the differentiation of monocytes towards both DC and inflammatory M1 macrophages, switching them into an anti‐inflammatory/regulatory M2 profile, a characteristic common to N‐hAMSC 33.

In this study, we have demonstrated that PE‐hAMSC are able to inhibit both CD4 and CD8 T‐cell proliferation, and to suppress the differentiation of monocytes towards DC and M1 macrophages, as described for N‐hAMSC 21. Previously, others have shown similar cytokine secretion profiles between hAMSC from PE and normal pregnancies 47. Although these results have to be confirmed by a larger sample size, overall, these data corroborate the hypothesis that there is no intrinsic impairment of the immunomodulatory features of hAMSC in PE. Our observations that PE‐hAMSC suppress Th1/Th2/Th17 polarization, and induce regulatory T and M2 cells, could possibly suggest their contribution to creating a microenvironment useful for a successful/healthy pregnancy. Interestingly, PE‐hAMSC have more pronounced capabilities of suppressing Th1 (IFN‐γ production) and inducing Treg formation when compared to N‐hAMSC. These findings suggest that PE‐hAMSC do not possess an intrinsic defect nor do they participate to the disease mechanism, but conversely, it seems that PE‐hAMSC could efficiently counteract the inflammatory environment that characterizes PE and, ultimately, help foetal survival.

## Conflicts of interest

The authors declare no conflicts of interest.
